# Measuring Surgical Outcomes in Cervical Spondylotic Myelopathy Patients Undergoing Anterior Cervical Discectomy and Fusion: Assessment of Minimum Clinically Important Difference

**DOI:** 10.1371/journal.pone.0067408

**Published:** 2013-06-24

**Authors:** Brenda M. Auffinger, Rishi R. Lall, Nader S. Dahdaleh, Albert P. Wong, Sandi K. Lam, Tyler Koski, Richard G. Fessler, Zachary A. Smith

**Affiliations:** 1 The University of Chicago, Section of Neurological Surgery, Chicago, Illinois, United States of America; 2 Northwestern University, Department of Neurological Surgery, Chicago, Illinois, United States of America; University of Louisville, United States of America

## Abstract

**Object:**

The concept of minimum clinically important difference (MCID) has been used to measure the threshold by which the effect of a specific treatment can be considered clinically meaningful. MCID has previously been studied in surgical patients, however few studies have assessed its role in spinal surgery. The goal of this study was to assess the role of MCID in patients undergoing anterior cervical discectomy and fusion (ACDF) for cervical spondylotic myelopathy (CSM).

**Methods:**

Data was collected on 30 patients who underwent ACDF for CSM between 2007 and 2012. Preoperative and 1-year postoperative Neck Disability Index (NDI), Visual-Analog Scale (VAS), and Short Form-36 (SF-36) Physical (PCS) and Mental (MCS) Component Summary PRO scores were collected. Five distribution- and anchor-based approaches were used to calculate MCID threshold values average change, change difference, receiver operating characteristic curve (ROC), minimum detectable change (MDC) and standard error of measurement (SEM). The Health Transition Item of the SF-36 (HTI) was used as an external anchor.

**Results:**

Patients had a significant improvement in all mean physical PRO scores postoperatively (p<0.01) NDI (29.24 to 14.82), VAS (5.06 to 1.72), and PCS (36.98 to 44.22). The five MCID approaches yielded a range of values for each PRO: 2.00–8.78 for PCS, 2.06–5.73 for MCS, 4.83–13.39 for NDI, and 0.36–3.11 for VAS. PCS was the most representative PRO measure, presenting the greatest area under the ROC curve (0.94). MDC values were not affected by the choice of anchor and their threshold of improvement was statistically greater than the chance of error from unimproved patients.

**Conclusion:**

SF-36 PCS was the most representative PRO measure. MDC appears to be the most appropriate MCID method. When MDC was applied together with HTI anchor, the MCID thresholds were: 13.39 for NDI, 3.11 for VAS, 5.56 for PCS and 5.73 for MCS.

## Introduction

Cervical spondylotic myelopathy (CSM) is the most common form of spinal cord dysfunction in the United States and the most prevalent cause of spinal cord injury in individuals older than 55 years [1]. Patient-reported outcome (PRO) questionnaires, such as Visual Analog Scale (VAS) [2], Neck Disability Index (NDI) [3], [4] and Short Form 36 (SF-36) [5], are often used for the evaluation of the clinical impact of cervical spine surgery on patients’ functional status and response to treatment. However, the numeric values provided by the analysis of these surveys usually lack direct clinical significance. The concept of minimum clinically important difference (MCID) has previously been used to determine the smallest change that is meaningful to patients [6]. Thus, it may help establish a critical threshold necessary to achieve treatment effectiveness.

Several anchor- and distribution-based approaches are available for MCID calculation [7]–[10]. A major limitation imposed by these methods is that different calculation approaches may yield a wide range of MCID threshold values [9], [11]. As a result, the choice of calculation method has direct implications on the evaluation of the treatment. Therefore, the comparison of different approaches is very important when analyzing the clinical impact of a specific therapy, since it can dictate the most representative MCID threshold for a given population. Thus far there is no consensus on the optimal MCID approach for the four common PRO measures used to evaluate patients undergoing cervical spine surgery: NDI, VAS, PCS and MCS.

Two previous studies have compared different MCID calculation methods in cervical spine patients. Carreon et al. in 2010 described MCID values for a heterogeneous population undergoing both anterior and posterior cervical spine fusion [12]. More recently, Parker et al. assessed anchor-based approaches in patients with cervical radiculopathy undergoing anterior cervical discectomy and fusion (ACDF) [13]. This latter study however, only had 3-month follow-up. No studies to date have evaluated the effectiveness of a specific therapy, such as ACDF, in a homogeneous population of myelopathic patients with long-term follow up. The goal of this study was to compare different anchor-based and distribution-based approaches for MCID calculation using NDI, VAS, PCS and MCS as PRO measures in CSM patients undergoing ACDF. Specifically, we aimed to determine which MCID thresholds and statistical methods represent the most clinically meaningful measure of surgical outcome following ACDF.

## Methods

### Patient Sample

Our prospectively collected spine surgery registry was retrospectively examined. Charts were reviewed of 169 consecutive patients undergoing ACDF between January 2007 and September 2012 by two surgeons at Northwestern Memorial Hospital. Only patients with CSM were included in this study. CSM was defined by the following signs and symptoms: corticospinal distribution deficits, atrophy of hand intrinsic muscles, hyperreflexia, presence of a Hoffman’s or Babinski reflex, spasticity or clonus, broad-based unstable gait, impairment of fine motor function, or bilateral arm paresthesia in the setting of appropriate radiographic findings. Inclusion criteria were: MRI confirmation of degenerative CSM disease and age over 18 years. Patients with trauma, infection or intracranial tumors, peripheral nerve disease as a cause of symptoms were excluded. Patients were also excluded if they did not complete PRO questionnaires preoperatively and at 1 year follow-up. A total of 30 patients met inclusion criteria.

All PRO questionnaires were completed by the patients either at the doctor’s office or at home and returned by mail. Institutional review board approval was received from the Northwestern University Research Subject Protection Program.

### Patient-Reported Outcomes

Four patient-reported outcome questionnaires were completed by patients preoperatively and 1 year after surgery: NDI [3], PCS and MCS from SF-36 [14], and VAS for neck pain [15]. Investigators not clinically involved with the patients assessed patient outcomes questionnaires. We define “change scores” as the difference between baseline and 1-year postoperative follow-up scores. The NDI is a 10-item patient survey that quantifies disability in patients suffering from neck pain. It has a maximum score of 50 with every item scored from 0–5; higher scores reflect increased disability [5], [16]. The SF-36 is a 36-item health questionnaire. Based on the reported values, two main scores can be calculated: PCS (physical component summary) and MCS (mental component summary). The SF-36 primarily evaluates patients’ social and physical function, general health, vitality and body pain. VAS relies on a self-assessment numerical scale that ranges from 0 to 10 for pain [10]. Zero signifies no pain, while 10 represents intolerable pain. Decreasing scores for NDI and VAS, and increasing values for the PCS and MCS components of the SF-36 imply improved functional status.

### Anchors

The health transition item (HTI) of the SF-36 was used as the anchor for derivations of anchor-based MCID calculations. The HTI refers to how the patient feels at the time of the questionnaire compared to one year ago. This is considered an appropriate independent anchor because is it not used in the scoring of MCS or PCS of the SF-36.

### Anchor-based and Distribution-based Approaches

We used five statistical methods for calculation of MCID for each of the above PRO scores. These include three previously reported anchor-based approaches: mean change, change difference, and receiver operating characteristic curve (ROC); and two distribution-based approaches: minimum detectable change (MDC) and standard error of measurement (SEM). “Mean change” stands for an MCID value that correlates with the average change in the patient cohort that exhibits small PRO variations. In this approach, the selection of groups of patients in different scales for MCID calculation is subjective. It depends on the number of levels in the original scale [17].

The “change difference” MCID approach aims to compare PRO score changes between two adjacent levels of a given scale [18]. In our case, it compares the difference in change scores of the patients that feel “minimally improved” and “minimally worse” for the anchor that was used in our MCID calculation. The “minimum detectable change” (MDC) is the smallest value that is above the measurement error within a 95% confidence interval (CI). MDC uses the standard error of measurement (SEM) for the calculation of an MCID with a 95% CI [19], [20].

The receiver operating characteristic (ROC) curve is a sensitivity- and specificity-based approach for calculation of MCID. When applied to PROs and used in conjunction with MCID, a sensitivity of 1 means that all true positive values have been identified (patient reports an improvement and MCID is above the therapeutic threshold). The inverse applies for a specificity value of 1 [21], [22]. The ROC curve ideally identifies the threshold for a PRO score while keeping the greatest sensitivity and specificity. The area under the ROC curve represents the probability that a PRO score will discriminate between improved and unimproved patients. The probability values range between 0.5 (probability of discrimination is the same as a coin toss) and 1 (accurately discriminates all patients) [9].

The “standard error of measurement” (SEM) estimates standard error in a repeated set of scores. It has a direct correlation with the reliability of the test. A change in score above the preoperative SEM values reflects a true change. In our analyses, SEM was defined as SD × (1 - *r*)^1/2^, where SD was the standard deviation of the baseline scores and *r* was the test-retest reliability coefficient [6], [23], [24]. A reliability of 0.90 was used for NDI [25], 0.95 for MCS, 0.92 for PCS [26], and 0.95 for VAS pain scales [27].

### Statistical Analyses

All statistical analyses were carried out in Prism 5 for Mac OS X version 5.0c (Graphpad Software Inc, La Jolla, CA) and STATA 11.1 (StataCorp, College Station, TX). Paired sample *t* tests were used to compare preoperative and 1-year postoperative scores. We used one-way analysis of variance with Bonferroni post hoc tests to compare change in outcome scores between groups classified according to responses to the anchor question. Values with p<0.05 were considered statistically significant.

## Results

Preoperative and 1 year postoperative PRO scores were collected from 30 patients. Mean age of patients at baseline was 57.53±12.98 years. 16 patients (53.33%) were female, 14 (47.66) were male. Mean body-mass index (BMI) was 27.98±5.67. 40% of the patients were either current or previous smokers (**Table 1**). 11 patients (36.66%) underwent single-level decompression and fusion, while 19 patients (63.33%) had multi-level decompression and fusion (**Table 2**).

**Table 1:** Patient’s overall characteristics at baseline.

Patient’s overall baseline characteristics	N (%) of cases or mean/SD values
No. of patients	30
Mean age (years)	57.53±12.98
% of females	53.33%
Mean BMI	27.98±5.67
Smoking history (%)	40%

Abbreviations: No., Number of Patients; %, Percentage of Patients; SD, Standard Deviation; BMI, Body Mass Index.

**Table 2:** ACDF population divided by surgical approach.

Surgical approach	Number of patients	% of patients
Single-level decompressionand fusion	11	36.66%
Multi-level decompressionand fusion	19	63.33%

Abbreviations: ACDF, Anterior Cervical Discectomy and Fusion.

The mean duration of surgery was 177.3±75.09 minutes, with an average hospital stay of 1.7±1.29 days. Estimated blood loss was 54.5±56.65 ml. No major complications occurred within 30 days of the index surgery. As a minor complication, one patient developed post-operative atrial fibrillation with rapid ventricular rate, which was promptly controlled medically. There were no readmissions, surgical sites infections, nor reoperations. The mean baseline, 1-year and change in PRO scores for NDI, VAS, PCS and MCS of the SF-36 survey are described in **Table 3**. All patients showed significant improvement for all physical PRO measures 1 year after surgery (p<0.01). Although, on average all patients experienced an improvement in their mental state post-surgery, such difference was not statistically significant. The mean changes between baseline and 1 year for NDI, VAS, PCS and MCS scores were −14.41±12.09, −3.34±2.67, 7.23±9.01, 0.38±8.02 respectively.

**Table 3:** Patient-reported outcomes - NDI, PCS, MCS and VAS – at baseline and 1 year post-surgery, and change in outcome scores.

Patient-reported outcomes	Baseline			1 Year			Change		
	Mean	SD	No.	Mean	SD	No.	Mean	SD	No.
**NDI**	29.24	13.94	30	14.82	11.81	30	−14.41	12.09	30
**VAS**	5.06	2.64	30	1.72	1.96	30	−3.34	2.67	30
**PCS**	36.98	7.13	29	44.22	9.71	29	7.23	9.01	29
**MCS**	47.82	7.93	29	48.20	7.54	29	0.38	8.02	29

Abbreviations: NDI, Neck Disability Index; PCS, Physical Component Summary of the Short Form of the SF-36; MCS, Mental Component Summary of the Short Form of the SF-36; VAS, Visual Analog Scale; SD, Standard Deviation; No., Number of Patients. Baseline means preoperative. Change means the difference between 1 year and baseline values.

The comparison of different anchor- and distribution-based approaches yielded a wide range of MCID threshold values for each PRO measure (**Table 4**). It varied from 2.00 to 8.78 for PCS, 2.06 to 5.73 for MCS, 4.84 to 13.39 for NDI, and 0.36 to 3.11 for VAS. When compared to the other four approaches, MDC appeared to be the most appropriate method for MCID calculation. The MDC approach generated a threshold of therapeutic improvement that was statistically greater than chance error from unimproved patients (>95% confidence interval (CI)). When this method was applied with the HTI anchor, the MCID thresholds were 5.56 for PCS, 5.73 for MCS, 13.39 for NDI and 3.11 for VAS.

**Table 4:** MCID threshold values for PCS, MCS, NDI, and VAS patient-reported outcome scores.

MCID Calculation Method	Patient-Reported Outcome Measure				
	PCS	MCS	NDI	VAS	
**Mean Change**	7.76	3.01	13	2.7	
**Change Difference**	8.78	5.13	5	0.36	
**MDC**	5.56	5.73	13.39	3.11	
**SEM**	2.00	2.06	4.83	1.12	

Abbreviations: NDI, Neck Disability Index; VAS, Visual Analog Scale; PCS, Physical Component Summary of the Short Form of the SF-36; MCS, Mental Component Summary of the Short Form of the SF-36; MCID, Minimum Clinically Important Difference; MDC, Minimum Detectable Change; SEM, Standard Error of Measurement. Anchor-based approaches: Mean Change, Change Difference and MDC. They were calculated based on the Health Transition Item of the SF-36 (HTI) as an anchor. Distribution-based approach: SEM.

**Figure 1:**
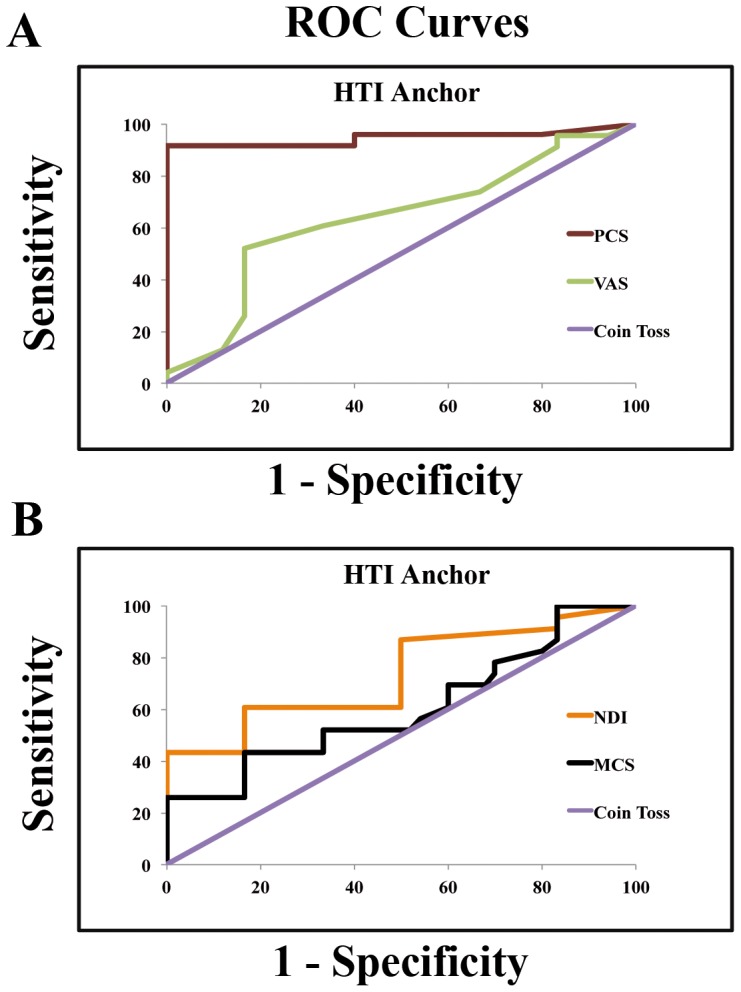
ROC curve plots comparing all four patient-reported outcome (PRO) measures collected in our dataset: NDI, VAS, PCS and MCS. All calculations were performed using the Health Transition Item of the SF-36 (HTI) as an anchor. (**A**) ROC curves comparing PCS and VAS PROs. The area under the curve for PCS and VAS, respectively, is 0.94 and 0.63. (**B**) ROC curves comparing NDI and MCS PROs. The area under the curve for NDI and MCS, respectively, is 0.67 and 0.57. Abbreviations: NDI, Neck Disability Index; PCS, Physical Component Summary of the Short Form of the SF-36; MCS, Mental Component Summary of the Short Form of the SF-36; VAS, Visual Analog Scale; ROC, Receiver Operating Characteristic Curve.

In order to evaluate which PRO was the most valid and responsive measure of therapeutic effectiveness in CSM patients undergoing ACDF, we used ROC curves to compare all four PRO measures (NDI, VAS, PCS and MCS) assessed in our study (**Figure 1**). The area under the curve (AUC) varied from 0.57 to 0.94, indicating that the ROC curve presented suitable accuracy on discriminating responders and nonresponders. The AUC for NDI, VAS, PCS and MCS was, respectively, 0.67, 0.63, 0.94 and 0.57. PCS was the PRO measure that seemed to be the most accurate discriminator of meaningful effectiveness (AUC of 0.94) and most responsive to post-operative improvement. The AUC for NDI, VAS and MCS was below the 0.7 threshold of considerable acceptance.

**Figure 2:**
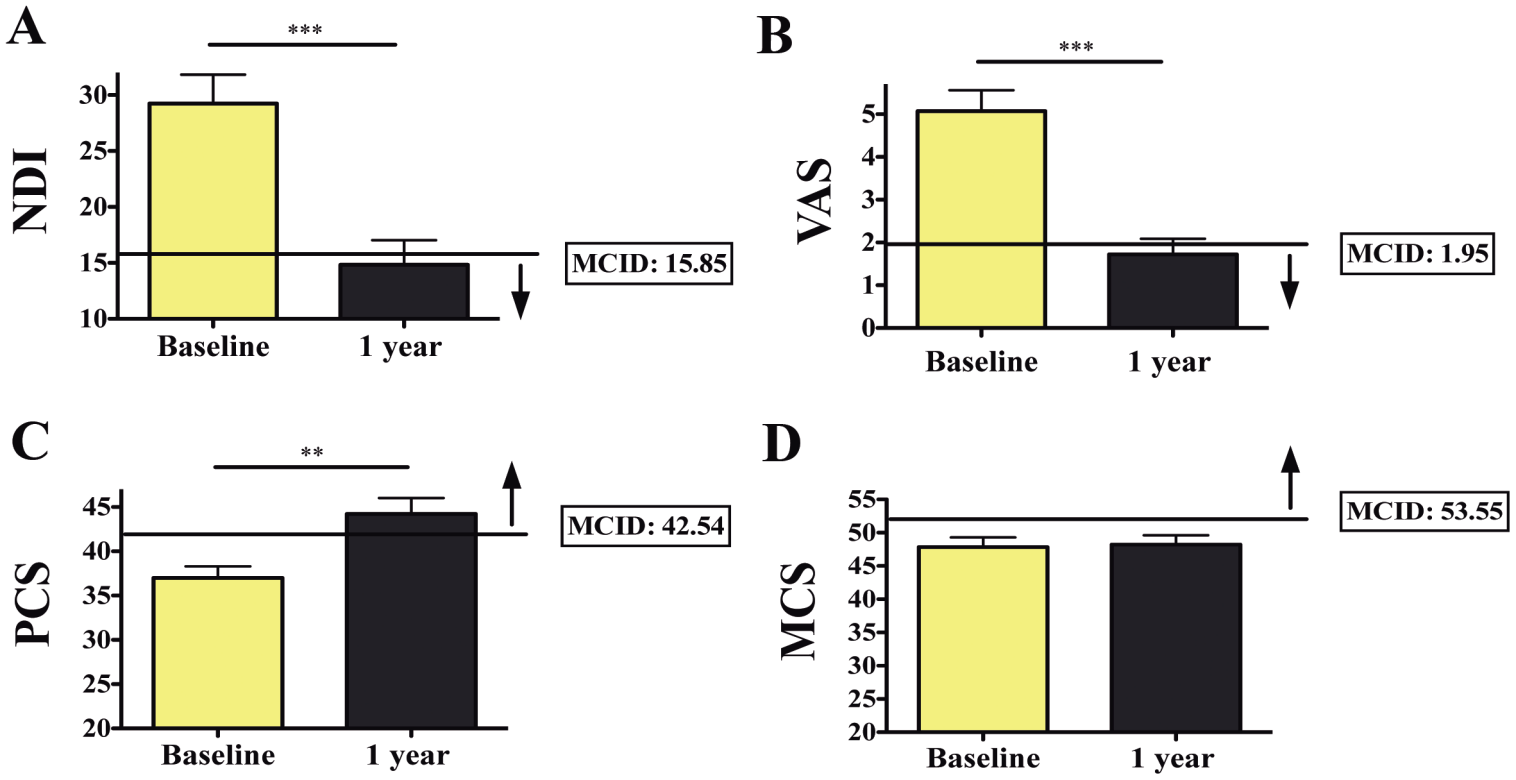
Mean baseline and 1-year PRO scores from patients undergoing surgery for anterior cervical discectomy and fusion (ACDF) and their respective MCID threshold values. (**A**) NDI, (**B**) VAS, (**C**) PCS PRO measures demonstrated patients’ clinically meaningful and statistically significant improvement 1 year post-surgery. (**D**) MCS values did not vary significantly. On average, all patients presented high MCS values (very good mental condition) at baseline and 1 year post-surgery. The overall mean change scores were: NDI −14.41±12.09 (p<0.001), VAS −3.34±2.67 (p<0.001), PCS 7.23±9.01 (p<0.01) and MCS 0,38±0.02 (p: 0.85). The MCID threshold score for each PRO measure was: NDI 15.85, VAS 1.95, PCS 42.54 and MCS 53.55. HTI was chosen as the anchor for the calculation of the MCID threshold values. MDC represents the chosen anchor-based approach. Abbreviations: NDI, Neck Disability Index; PCS, Physical Component Summary of the Short Form of the SF-36; MCS, Mental Component Summary of the Short Form of the SF-36; VAS, Visual Analog Scale; MCID, Minimum Clinically Important Difference; PRO, Patient-Reported Outcome; **, p<0.01; ***, p<0.001.

On average, all patients in our study achieved the desired MCID threshold value for the PROs that assessed the patient’s physical status (NDI, VAS and PCS) (**Figure 2**). In general, the CSM patients treated with ACDF presented clinically meaningful and statistically significant improvement based on a subjective external anchor (HTI). Although the patients presented an average increase in MCS scores, this same group of patients did not achieve the desired MCID threshold for the MCS PRO.

## Discussion

In this study, we analyzed different MCID approaches for our patient population of adults with CSM treated with ACDF with 1-year follow-up.

### Analysis of our Population and Comparison with Other Studies

The motivation for this study was to evaluate different MCID calculation approaches with the goal of identifying the most clinically meaningful and statistically significant MCID value for different PRO measures in patients undergoing ACDF for cervical spondylotic myelopathy. Other studies have evaluated different anchor- and distribution-based approaches, such as mean change [7], [28], average change [7], MDC [7], [12], [28], sensitivity- and specificity-based approaches (ROC curves) [7], [12] and SEM in patients undergoing spine surgery [12]. However, an optimal MCID threshold value or best MCID calculation method has not been established for myelopathic patients undergoing ACDF.

In contrast with previous studies on MCID for spine surgery [12], [13], we analyzed a homogeneous population in which all patients were diagnosed with cervical spondylotic myelopathy and treated with a specific surgical intervention (ACDF). The assessment of such a homogeneous population allows for an accurate investigation of the impact of a specific therapeutic intervention on patient quality of life.

### Choice of MCID Calculation Approach

Similar to other reports we compared two subsets of patients: those who rated themselves as “improved” (responders) and those whose rated themselves as “about the same” (nonresponders) [6], [10], [18], [29]. These calculations were based on HTI, a well-established subjective external anchor used in a number of previous studies [7], [12]. In our findings, MDC was most correlated with patient outcomes and allowed for the best statistical prediction of clinical improvement. It was consistently greater than measurement error (allowing for reliable interpretation of true change in treatment effectiveness), and it corresponded well to the patient perception of therapeutic improvement. For analogous reasons, additional reports have also identified MDC as the most reliable MCID calculation method compared to other approaches [7], [10], [28], [30]. Our MDC values are also in keeping with other previously described MCID thresholds [12], [18].

### Patient Overall Improvement

For each PRO measure used in our study (NDI, VAS and PCS), the mean reduction in postoperative score was greater than the MCID threshold; this reflects clinically and statistically significant functional improvement in our patient populace. Patients with lower baseline scores showed greater improvements in physical PRO scores at one-year follow-up. In contrast, improvement in mental composite scores did not meet statistical significance. One possible explanation for this finding is that our patient population presented with high preoperative MCS scores compared to previous studies (47.82±7.93), reflecting high premorbid mental health and emotional functioning. As such, there may have been less margin for improvement in MCS.

### Limitations of the Study

The present study has limitations that may affect optimal analysis. First, our sample size is small and thus our study may not be adequately powered to identify all statistically significant changes in PRO scores. In addition, we restricted the population under study to patients with a single diagnosis with one specific intervention. As a result, it may be difficult to assess if some of the variations in MCID thresholds seen in this study are actually due to differences inherent to CSM, anterior fusion, or statistical artifact. This limits the generalizability of our results.

Second, the lack of an objective external anchor may limit our ability to identify the most representative MCID calculation method. Subjective external anchors, the current mainstay for MCID computation, use a single-item self-report (HTI) to evaluate patient’s overall improvement in PRO scores [7], [8], [10], [12], [13]. This becomes statistically problematic since subjective anchors use one self-report score to validate another self-report score. Behavioral measures, such as health care use, medications and return to work, have been tested as possible objective external anchor-based approaches, however none of these measures have been validated [31], [32]. As such, in the absence of objective measures of functional outcome, current studies on MCID are limited to use of subjective anchors.

### Conclusions

In our examination of CSM patients treated with ACDF, MCID threshold values were highly variable depending on the calculation method. The MDC approach was shown to be the most clinically relevant and statistically significant technique for MCID calculation. The threshold of improvement of MDC values was statistically greater than the chance of error from unimproved patients. Taking into account the wide range of values for MCID calculation obtained from the comparison of different approaches, MDC together with the HTI anchor appears to be the most appropriate MCID method. In addition, PCS seemed to be the most valid and responsive measure of effectiveness for CSM patients undergoing ACDF surgery.
